# Carcinosarcoma of the gallbladder—an exceedingly rare tumour

**DOI:** 10.1259/bjrcr.20160019

**Published:** 2016-11-02

**Authors:** João Cruz, António P Matos, Jorge O Neta, Miguel Ramalho

**Affiliations:** ^1^Department of Radiology, Hospital Garcia de Orta, Almada, Portugal; ^2^Department of Pathology, Hospital Garcia de Orta, Almada, Portugal

## Abstract

Carcinosarcoma of the gallbladder (CSGB) is an extremely rare tumour that presents variable proportions of malignant epithelial and mesenchymal elements. Preoperative diagnosis of CSGB is challenging owing to its non-specific clinical presentation and imaging findings. The final diagnosis requires histopathological confirmation of both the epithelial and mesenchymal components. Owing to the low incidence and poor prognosis of this tumour, it is essential to gather all the individual experience-based information. We report a case of a 52-year-old female who presented with right upper abdominal pain and vomiting for 2 weeks and a painless palpable mass on the right upper quadrant of the abdomen. Imaging studies showed a complex gallbladder mass, which was pathologically confirmed to be CSGB. To our knowledge, this is the first CSGB presented from a radiological perspective. A short literature revision of CSGB is provided.

## Summary

Carcinosarcoma of the gallbladder (CSGB) is an extremely rare tumour that presents variable proportions of malignant epithelial and mesenchymal elements. Preoperative diagnosis of CSGB is challenging owing to its non-specific clinical presentation and imaging findings. The final diagnosis requires histopathological confirmation of both epithelial and mesenchymal components. Owing to the low incidence and poor prognosis of this tumour, it is essential to gather all the individual experience-based information. We report a case of a 52-year-old female who presented with right upper abdominal pain and vomiting for 2 weeks and a painless palpable mass on the right upper quadrant of the abdomen. Imaging studies showed a complex gallbladder mass, which was pathologically confirmed to be CSGB. To our knowledge, this is the first CSGB presented from a radiological perspective. A short literature review of CSGB is provided.

## Clinical presentation

A 52-year-old female presented to the emergency department with right upper abdominal discomfort and vomiting for 2 weeks. Her personal and family history were unremarkable. On physical examination, there was a palpable painless hard mass on the right upper quadrant of the abdomen. The remaining physical examination was unremarkable.

## Investigations and imaging findings

Serum liver enzyme levels were slightly elevated [alanine transaminase level, 118 U l^−1^ (normal level, < 60 U l^−1^); aspartate transaminase level, 41 U l^−1^ (normal level, < 40 U l^−1^)]. The remaining laboratory tests were normal, including total bilirubin and alkaline phosphatase values, as well as tumour markers—carcinoembryonic antigen, cancer antigen (CA) 19-9, CA 125 and α-fetoprotein. Chest X-ray was unremarkable.

**Figure 1.**
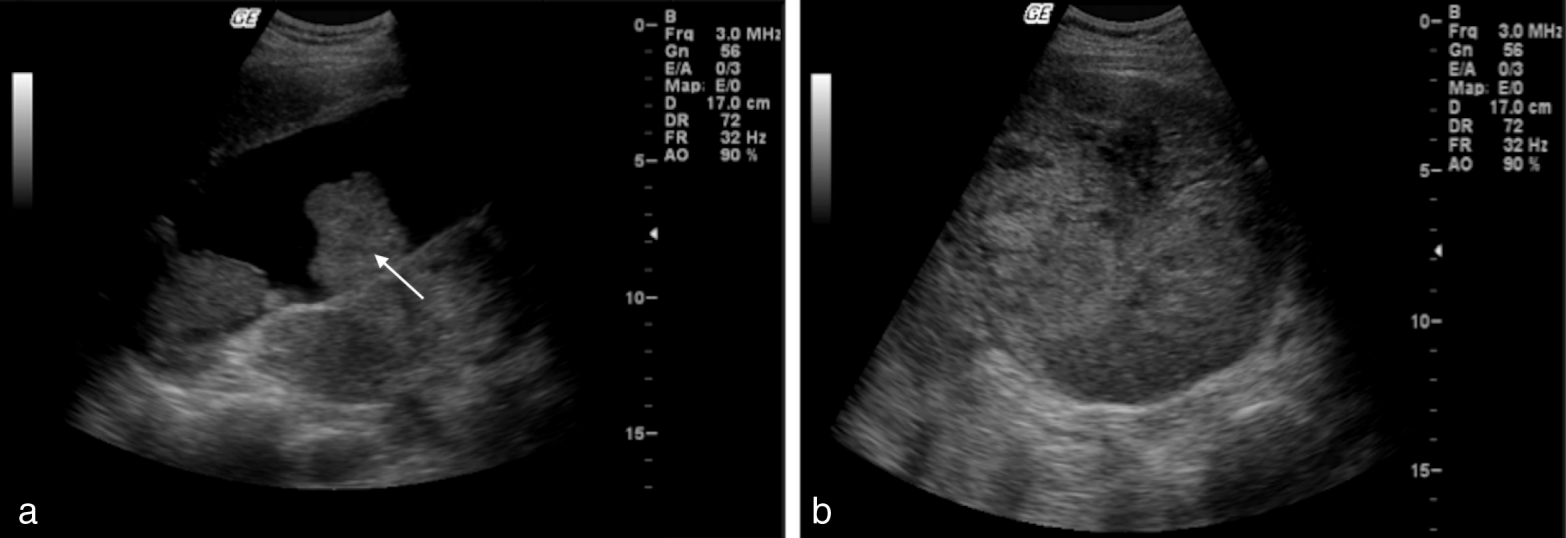
Abdominal ultrasonography images obtained at admission using a 5-3 MHz curvilinear probe show a distended gallbladder (a) with diffusely thickened wall and intraluminal polypoid lesions (arrow, a) in continuity with an exophytic, expansile, heterogeneous predominantly solid lesion (b).

Abdominal ultrasound imaging was performed, which showed a 17 cm solid mass in the right upper quadrant, inferior to the gallbladder. The gallbladder showed gallstones, polypoid lesions and a thickened wall adjacent to the aforementioned expansile mass (Figure 1).

**Figure 2.**
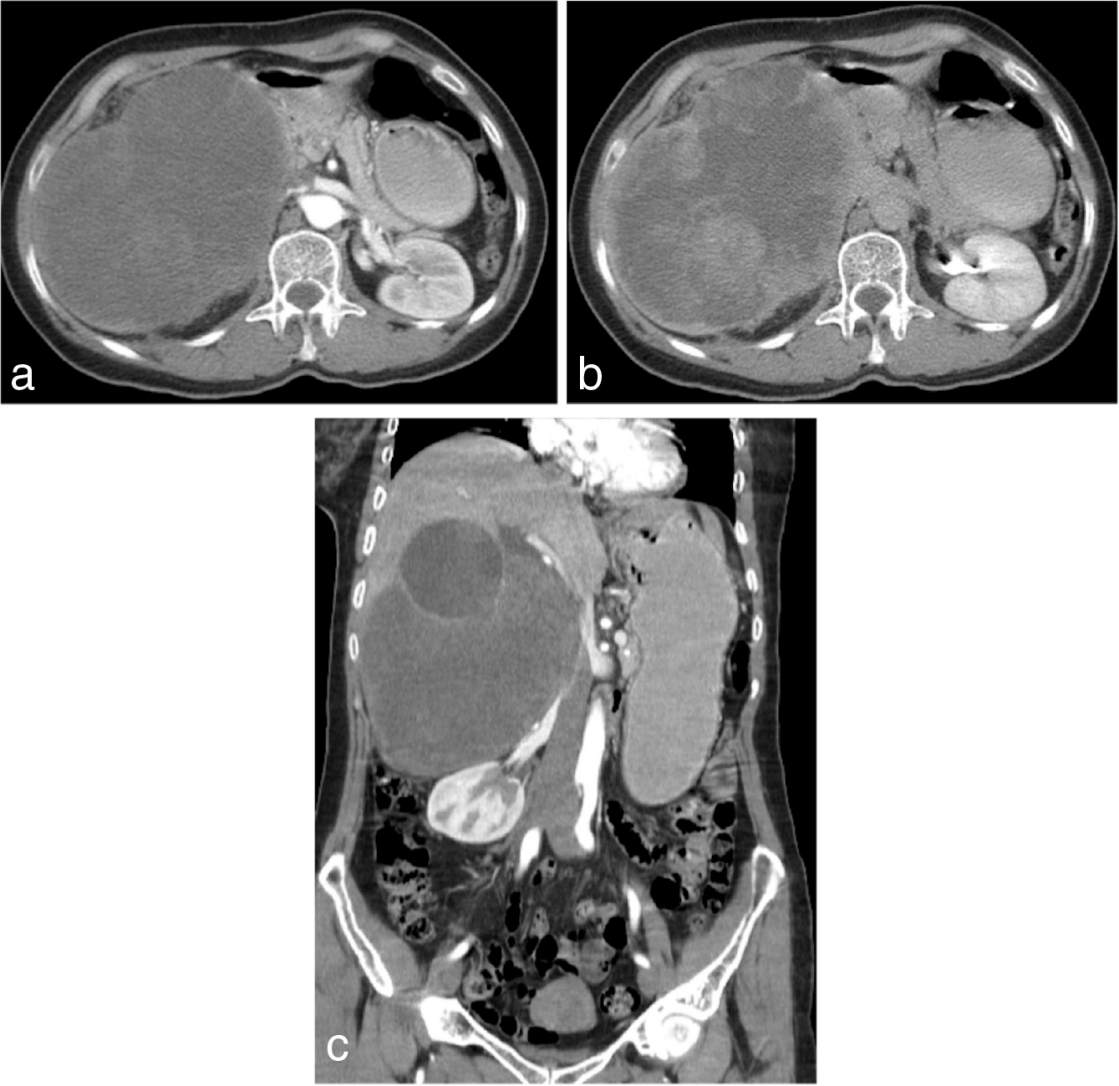
MDCT images obtained at admission. Transverse iodinated contrast-enhanced MDCT at the level of the left renal hilum in the arterial (a) and interstitial (b) phases showed a heterogeneous, expansile low soft tissue density mass abutting the inferior surface of the gallbladder and the right hepatic lobe, and compressing the hepatic hilum. It is hypovascular (a) with progressive enhancement on later phases (b). Coronal reformatted contrast-enhanced MDCT image of the abdomen and pelvis demonstrates an enlarged and distended gallbladder with heterogeneous filling (c). MDCT, multidetector CT.

A triple-phase contrast-enhanced multidetector CT (MDCT) scan (Figure 2) was performed for further evaluation. An enlarged and distended gallbladder with heterogeneous content was depicted. Contiguous to its inferior thickened wall, a heterogeneous, hypodense soft tissue expansile mass of size 17 × 12.5 cm was seen. This mass was hypovascular, with progressive enhancement on later phases. The mass abutted the inferior surface of the right hepatic lobe and compressed the hepatic hilum, causing mild dilatation of intrahepatic bile ducts. No intralesional calcifications were depicted.

**Figure 3.**
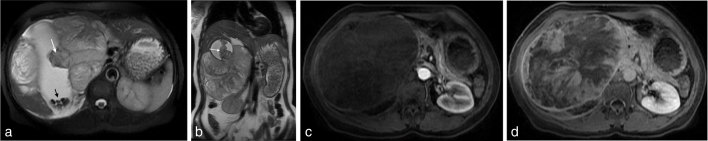
Transverse fat-suppressed single-shot fast spin-echo *T*_2_ weighted image (a) and coronal non-fat-suppressed single-shot fast spin-echo *T*_2_ weighted image (b) show a distended gallbladder with intraluminal polypoid lesions (arrows, a, b) in continuity with an exophytic expansile lesion occupying the right upper quadrant. This mass appears heterogeneous and with moderately high *T*_2_ signal intensity. Transverse gadolinium-enhanced fat-suppressed *T*_1 _weighted three-dimensional gradient-echo MRI obtained through the abdomen at the arterial (c) and interstitial (d) phases show the hypovascular nature (c) with progressive enhancement over time (d). Note the presence of gallbladder calculi, easily recognized on *T*_2_ weighted image (arrowhead, a).

A gadolinium-enhanced MRI (Figure 3) was requested 2 days later for further characterization. There was a better perception of contrast enhancement and the gallbladder origin of the large exophytic lesion. The mass exhibited low signal intensity on *T*_1_ weighted images and heterogeneous moderately high signal on *T*_2_ weighted images, showing what appeared to be numerous thickened septa. Enhancing polypoid lesions in the gallbladder were confirmed. The MRI showed no definite signs of invasion of the surrounding structures, despite a substantial displacement of the hepatic hilum, inferior vena cava, duodenum, pancreas, hepatic angle of the colon and right kidney. The right adrenal gland was not confidently identified. There were no liver lesions.

## Differential diagnosis

The differential diagnosis of a right upper quadrant tumour includes tumours originating from the liver, gallbladder, pancreas, duodenum, adrenal glands or kidneys. The MRI findings of gallbladder wall thickening, polyps and cleavage of all major locoregional structures suggested a gallbladder origin. Primary gallbladder malignancies were considered. In our opinion it would be extremely unlikely for a benign neoplastic process of the gallbladder, such as an adenomatous polyp, to present as a large exophytic mass, as seen in our case. Primary malignancy of the gallbladder was the most probable diagnosis. Primary malignant lymphomas of the gallbladder are extremely rare tumours and tend to present as a solid and bulky mass in the gallbladder or an irregular wall thickening.^1^ Lymphadenopathies and an intact mucosa appear to be a characteristic finding of this tumour, which were not seen in our case.

Malignancies of epithelial origin, including adenocarcinoma [gallbladder carcinoma (GBC)] and CSGB, were considered and are discussed ahead (Discussion section).

Adrenal origin, including adrenal primary malignancy (*i.e.*, adrenal carcinoma) or metastasis, could not be ruled out as there was no definite identification of the right adrenal gland. It would be unlikely for a primary adrenal malignancy to extend up to the gallbladder without invasion of any other organ, especially the kidneys. Furthermore, primary adrenal carcinoma usually manifests as a large mass, frequently with areas of necrosis and haemorrhage, which were not seen in our case, and sometimes with variable lipid content. Moreover, distant metastases are frequently found at presentation. On the other hand, adrenal metastasis was unlikely in a patient without a known primary malignancy or any other metastatic involvement. Additionally, the more common adrenal metastases usually manifest as solid lesions with central necrosis, which was not seen in our case.

## Treatment

**Figure 4.**
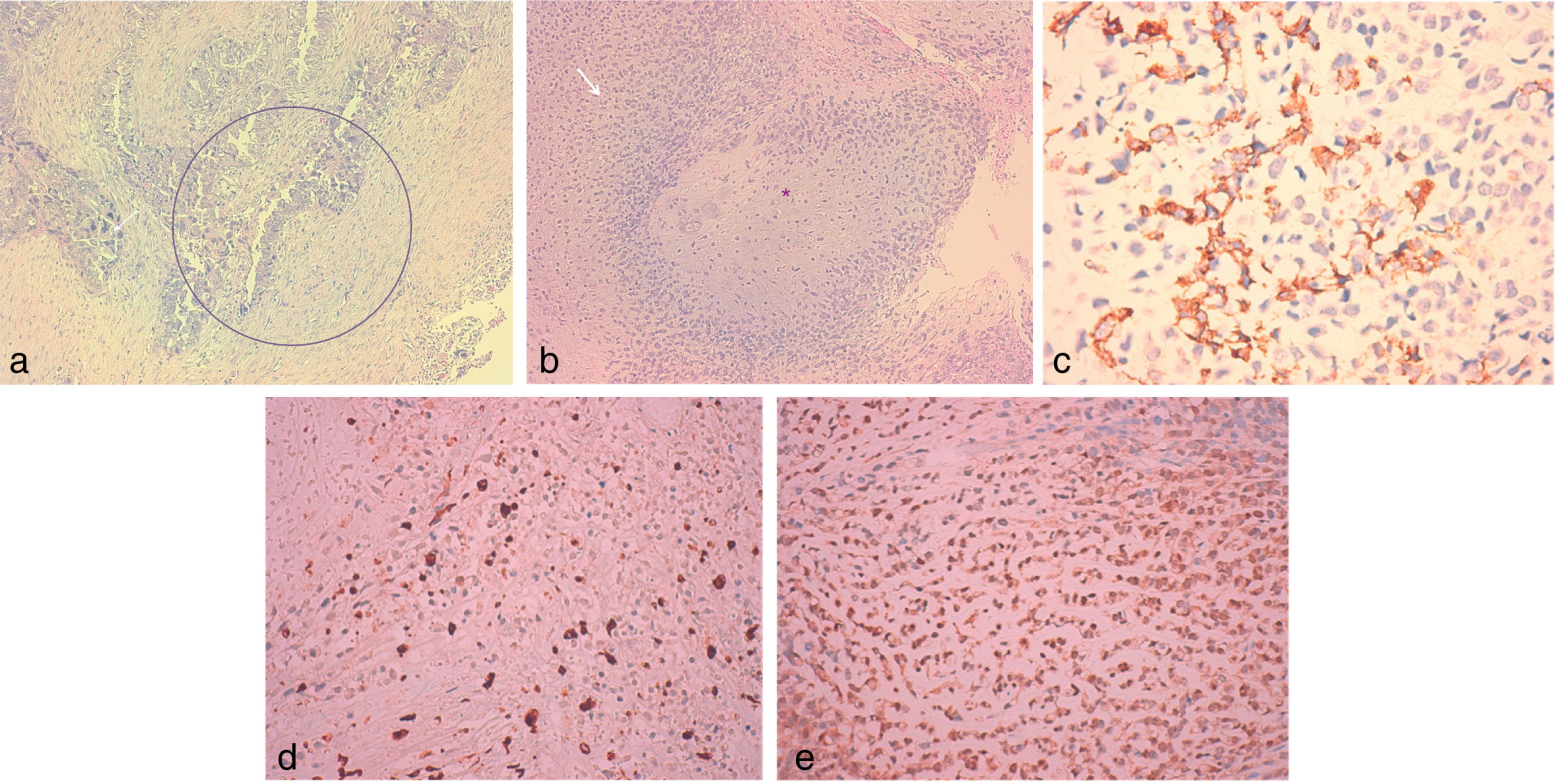
Microscopic examination of the excised tumour demonstrating the histological components of adenocarcinoma and sarcoma. (a) Infiltrative proliferation with epithelial phenotype and glandular architecture (circle) with moderate/accentuated cellular atypia and pleomorphic cells (haematoxylin and eosin staining, 10×); (b) interface between poorly differentiated epithelial phenotype of invasive carcinoma (arrow) and mesenquimatous component of sarcoma with atypical chondroid differentiation (asterisk) (haematoxylin and eosin staining, 10×); (c) immunostaining of the epithelial component (cytokeratin AE1/AE3, 40×); (d) immunoexpression on chondroid/mesenquimatous component (vimentine, 40×); (e) immunoexpression on chondroid/mesenquimatous component (S100, 40×).

A laparotomy was performed that revealed a large mass with gallbladder origin and exophytic growth. The mass was excised and an extended cholecystectomy, including minor hepatic resection near the gallbladder fossa, and lymphadenectomy were performed. There were no signs of invasion of the hepatic pedicle and particularly the proximal intrahepatic and common bile ducts. Pathological analysis revealed a moderately differentiated adenocarcinoma that invaded the entire wall of the gallbladder with an exophytic mass component. Extensive sarcomatous areas of cartilaginous differentiation, positive for cytokeratin and vimentin, were present (Figure 4). Demonstration of these two histological components was consistent with CSGB of the gallbladder.

## Outcome and follow-up

**Figure 5.**
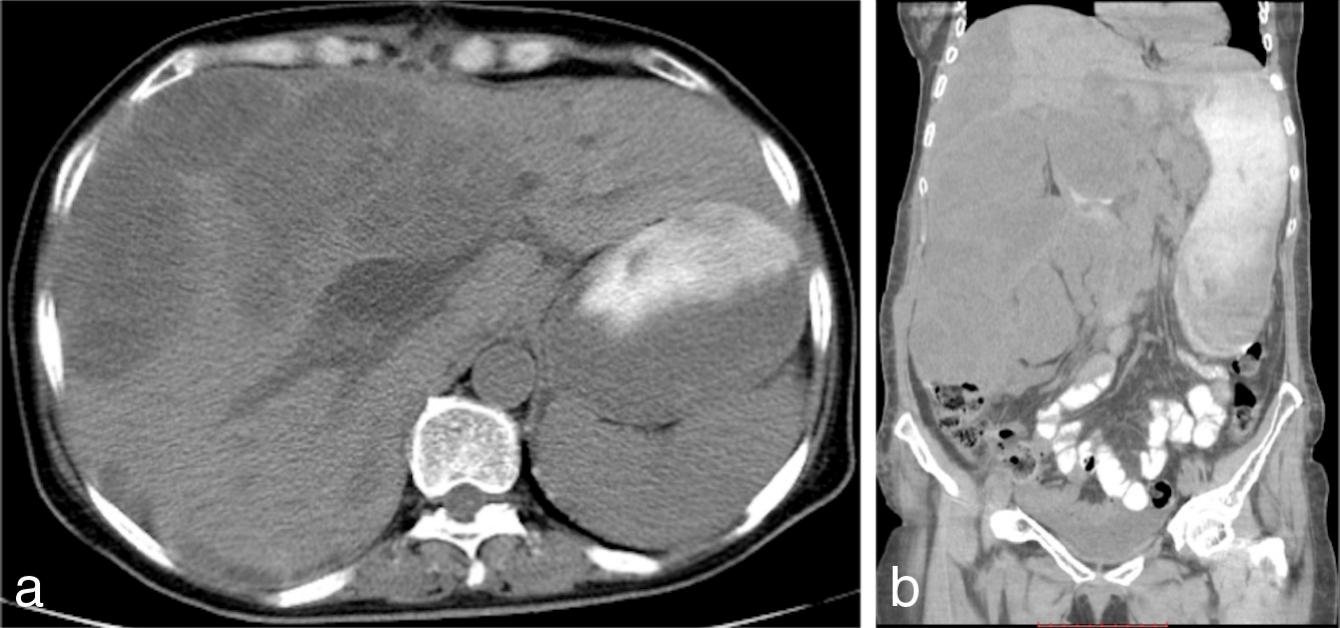
Axial (a) and coronal reformatted (b) images of non-enhanced multidetector CT show the presence of multiple hypodense lesions in the left hepatic lobe, as well as multiple nodular, solid hypodense intra-abdominal lesions compatible with recurrence of the resected carcinosarcoma of the gallbladder.

The patient was discharged 20 days after surgery. The patient was readmitted 26 days after discharge owing to abdominal distension and pain. An MDCT scan was performed (Figure 5) showing locoregional recurrence of the disease. The oncology team considered the patient clinically unsuitable for initiating chemotherapy and recommended palliative care. The patient died 5 days later. Considering the immediate abdominal spread, we believe that earlier histopathological evaluation and thus earlier initiation of chemotherapy would not have changed the course of the disease.

## Discussion

To our knowledge, this is the first case of CSGB reported from a radiological perspective. We believe this case is especially illustrative, as it shows the ultrasound, MDCT and MRI findings of this rare neoplasm, and also its recurrence.

Gallbladder cancer is relatively rare in the USA and most western European countries, with an incidence of approximately 1.2 per 100,000 persons, the vast majority being adenocarcinomas.^2,3^ CSGB are exceedingly rare tumours (< 1% of the gallbladder malignancies),^4^ first described by Karl Landsteiner in 1907.^5^ The diagnosis of CSGB relies on the concomitant demonstration of variable proportions of malignant epithelial (carcinoma) and mesenchymal (sarcoma) elements.^6,7^ The occurrence of this malignancy in other organs, such as the uterus, lungs, oesophagus and kidneys, is rare but more frequent than in the gallbladder.^4^

Preoperative diagnosis of CSGB is very difficult by imaging. Radiology literature describing the appearance of CSGB is remarkably scarce. It appears that CSGB is not associated with specific radiological signs. The main differential diagnosis is GBC, which tends to present in infiltrative or papillary forms. The infiltrative pattern may present as focal or diffuse asymmetric wall thickening in 20–30% of cases.^2^ It is common for infiltrative tumours to invade the subserosal plane into the liver. On the other hand, papillary tumours usually show a polypoid cauliflower-like appearance that tends to fill or replace the gallbladder lumen and may be present in 40–65% of cases.^2^ In 15–25% of cases, the initial detection of GBC appears as an intraluminal polypoid lesion with or without a thickened implantation base.^2^ In our case, CSGB showed no macroscopic tumour infiltration into the liver and, despite the presence of polypoid lesions, the mass was essentially exophytic, not filling or replacing the gallbladder lumen. Interestingly, in a series report of 17 uterine carcinosarcomas, 53% of cases showed exophytic growth, while none showed invasive growth.^8^ On MRI, CSGB appeared iso- to hypointense on *T*_1_ weighted images, without areas of high signal intensity that could be related with intralesional haemorrhage; and moderate heterogeneous hyperintensity on *T*_2_ weighted images. The moderately high signal on *T*_2_ weighted images, with a heterogeneous septated appearance, might be the strongest discriminator, as it resembles the appearance of visceral sarcomas, such as liver sarcomas,^9^ which could be regarded as a relatively distinctive feature of these tumours owing to its mesenchymal component. This high *T*_2_ signal intensity is very unlikely to be seen in GBC, which tends to appear with a more homogeneous and intermediate-to-low *T*_2_ signal intensity.^10^ Tanaka et al^8^ showed that, in 88% patients with uterine carcinosarcomas, more than half of the tumours showed higher signal intensity than the outer myometrium, which usually demonstrates an intermediate *T*_2_ signal intensity.^8^ The identification of speckled calcification within the tumour may also suggest the diagnosis of ossifying carcinosarcoma.^4,5^ In our case, despite cartilaginous differentiation, calcifications were not seen on the MDCT scan. Moreover, large GBC may show hypervascular rim enhancement on the arterial dominant phase images,^3^ which was not seen in our case.

The final diagnosis requires pathological evaluation, including immunohistological staining demonstrating the epithelial component as positive for cytokeratin and the mesenchymal component as positive for vimentin.^5,6^ In the majority of carcinosarcomas, the epithelial component consists of adenocarcinoma, and less often squamous cell carcinoma. The mesenchymal component consists of undifferentiated stellate and spindle-shaped cells that are occasionally accompanied by various proportions of heterogeneous elements, including chondrosarcoma, osteosarcoma, rhabdomyosarcoma and leiomyosarcoma.^4,6^

Similar to GBC, CSGB shows a female predominance (female to male ratio is 2–5 : 1)with reported mean age at diagnosis of 66.5–72 years.^5,11^ CSGB usually presents with non-specific symptoms and signs, similar to those of GBC, including right upper abdominal pain, loss of appetite, weight loss, general fatigue, jaundice and vomiting. Tumour markers such as α-fetoprotein, carcinoembryonic antigen and CA19-9 are non-specific.^5–7^

CSGB is a very aggressive malignancy that usually presents with large size and spreads by invading the adjacent organs, and haematogenous and lymph node metastases. Regional, retroperitoneal and para-aortic lymph nodes may be involved.^5–7^ The prognosis is poor owing to the usual presence of locally advanced disease at diagnosis, with a reported mean survival of 2.9–6 months in general, and a 1- and 5-year survival rate of 19 ± 5% and 16 ± 5%, respectively.^5^ Those who present with symptoms suggestive of acute cholecystitis usually have early stage disease and a better prognosis, with a 5-year survival rate of 88.9% after curative resection, when the invasion is restricted to the muscularis propria.^4,5^

Some studies suggest tumour size as a major prognostic factor (cases with tumours < 5 cm had a longer survival). They also indicated that the presence of gallstones, epithelial and mesenchymal component types, age and sex were of little prognostic value.^6,11^

Owing to the sparse literature and poor prognosis, therapeutic interventions have not been well defined yet. Surgical resection remains the first line of management but the poor surgical outcomes and the fact that conventional chemo- and radiotherapy have not proven to be successful may suggest that new adjuvant strategies are warranted.^4–7^ Recently, adjuvant chemotherapy using new drug agents has been considered as the standard therapeutic option following surgical resection.^4,7^

## Conclusions

The low incidence and poor prognosis of this tumour makes it essential to gather all the individual experience-based information in an attempt to define possible radiological features that may suggest preoperative diagnosis. MRI may be the imaging modality of choice, as the mesenchymal component may show relative distinctive characteristics, unlikely to be present in other gallbladder tumour types. In some cases, the detection of speckled calcifications on MDCT scans may also suggest the correct diagnosis.

## Learning points

1. Carcinosarcomas of the gallbladder are rare tumours that present variable proportions of malignant epithelial and mesenchymal elements.
2. Gallbladder carcinosarcoma usually presents with non-specific symptoms and signs, similar to those of adenocarcinoma.
3. Gallbladder carcinosarcoma usually presents as a large mass. The mesenchymal component may show distinctive characteristics on MRI.
4. In some cases, the detection of speckled calcifications on MDCT scan may suggest the diagnosis.
5. Surgical resection remains the first line of management, with poor prognosis owing to the aggressive behaviour of the tumour and the presence of locally advanced disease at diagnosis.
6. As malignancy tends to spread immediately to the abdomen, a different surgical technique may be required to improve the prognosis.

## Consent

Informed consent to publish this case (including images and data) was obtained and is held on record.
